# Transmembrane Domains of Highly Pathogenic Viral Fusion Proteins Exhibit Trimeric Association *In Vitro*

**DOI:** 10.1128/mSphere.00047-18

**Published:** 2018-04-18

**Authors:** Stacy R. Webb, Stacy E. Smith, Michael G. Fried, Rebecca Ellis Dutch

**Affiliations:** a Department of Molecular and Cellular Biochemistry, University of Kentucky, Lexington, Kentucky, USA; Boston University School of Medicine

**Keywords:** SARS, Ebola virus, fusion protein, influenza, rabies, transmembrane domain, virus

## Abstract

Enveloped viruses require viral fusion proteins to promote fusion of the viral envelope with a target cell membrane. To drive fusion, these proteins undergo large conformational changes that must occur at the right place and at the right time. Understanding the elements which control the stability of the prefusion state and the initiation of conformational changes is key to understanding the function of these important proteins. The construction of mutations in the fusion protein transmembrane domains (TMDs) or the replacement of these domains with lipid anchors has implicated the TMD in the fusion process. However, the structural and molecular details of the role of the TMD in these fusion events remain unclear. Previously, we demonstrated that isolated paramyxovirus fusion protein TMDs associate in a monomer-trimer equilibrium, using sedimentation equilibrium analytical ultracentrifugation. Using a similar approach, the work presented here indicates that trimeric interactions also occur between the fusion protein TMDs of Ebola virus, influenza virus, severe acute respiratory syndrome coronavirus (SARS CoV), and rabies virus. Our results suggest that TM-TM interactions are important in the fusion protein function of diverse viral families.

**IMPORTANCE** Many important human pathogens are enveloped viruses that utilize membrane-bound glycoproteins to mediate viral entry. Factors that contribute to the stability of these glycoproteins have been identified in the ectodomain of several viral fusion proteins, including residues within the soluble ectodomain. Although it is often thought to simply act as an anchor, the transmembrane domain of viral fusion proteins has been implicated in protein stability and function as well. Here, using a biophysical approach, we demonstrated that the fusion protein transmembrane domains of several deadly pathogens—Ebola virus, influenza virus, SARS CoV, and rabies virus—self-associate. This observation across various viral families suggests that transmembrane domain interactions may be broadly relevant and serve as a new target for therapeutic development.

## Abstract (precis)

Many important human pathogens are enveloped viruses that utilize membrane-bound glycoproteins to mediate viral entry. Factors that contribute to the stability of these glycoproteins have been identified in the ectodomain of several viral fusion proteins, including residues within the soluble ectodomain. Although it is often thought to simply act as an anchor, the transmembrane domain of viral fusion proteins has been implicated in protein stability and function as well. Here, using a biophysical approach, we demonstrated that the fusion protein transmembrane domains of several deadly pathogens—Ebola virus, influenza virus, SARS CoV, and rabies virus—self-associate. This observation across various viral families suggests that transmembrane domain interactions may be broadly relevant and serve as a new target for therapeutic development.

## OBSERVATION

Membrane fusion is a critical event in the life cycle of enveloped viruses. Fusion of the viral envelope with the target cell membrane is mediated by the viral fusion protein. The trimeric fusion protein undergoes a dramatic structural rearrangement from its metastable prefusion conformation to the postfusion conformation after triggering by an event like receptor binding or a pH change. Factors that contribute to prefusion stability have been identified in the ectodomain of several fusion proteins, including residues within the stalk domain (1–4). Though often thought to act as just an anchor, the fusion protein transmembrane domain (TMD) has been implicated in protein stability and function. The influenza virus fusion protein, hemagglutinin (HA), was engineered to replace the TMD with a lipid anchor, glycosyl-phosphatidylinositol (GPI), to elucidate the role of the TMD. The GPI-HA protein was unable to efficiently promote fusion, and the TMD was shown to be essential for pore enlargement, implicating the TMD in protein function (5, 6). Additional studies demonstrated that specific HA TMD residues are important for the protein’s oligomeric state and function (7–9). Furthermore, prefusion crystal structures of parainfluenza virus 5 (PIV5) fusion protein (F), HIV gp41, and Hendra virus (HeV) F required the addition of a trimeric coiled-coil for prefusion stabilization, essentially acting to replace the TMD (10–12). This requirement across viral families suggests that TM-TM interactions are broadly relevant.

**FIG 1:**
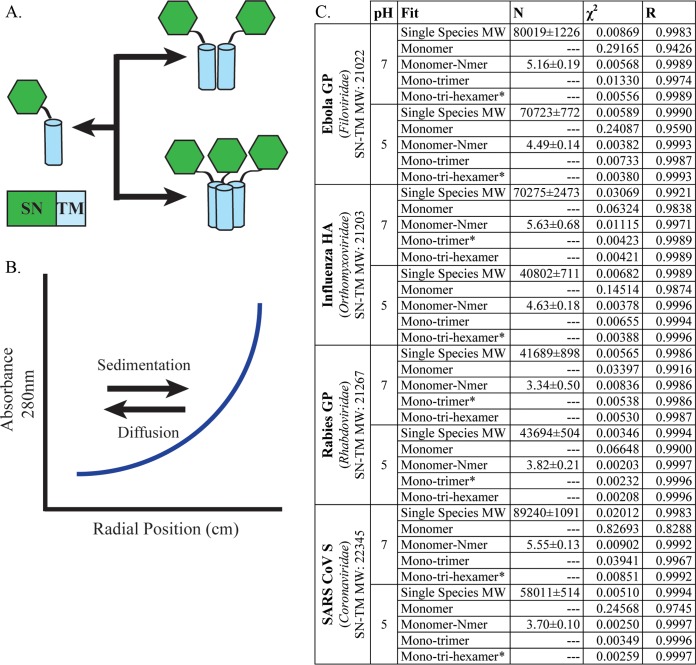
Parameters for SE-AUC. (A) Chimeric proteins were designed for SE-AUC, in which the TMD of interest was fused with the protein staphylococcal nuclease (SN). The addition of SN aids in purification and increases the extinction coefficient, which aids data collection in absorbance-based assays. (B) SE-AUC is based on the premise that, upon centrifugation, the species present will eventually reach an equilibrium wherein species of higher mass (such as a higher oligomeric state) will sediment, while species of lower mass will diffuse. The resulting spectra represent an equilibrium in which higher-molecular-weight species have a higher absorbance. The absorbance data can be fit using the following equation: $A\left(r\right)=\sum_{\text{species}}\alpha_{n}\;\exp\left[\sigma_{n}\left(r^{2}-r_{0}^{2}\right)\right]+\xi$, where *A* represents the total absorbance of the solution at radial position (*r*), while *α*_*m*,0_ and *α*_*m*/*t*,0_ represent the monomer (*m*) and monomer/trimer (*m*/*t*) absorbance, respectively, as the reference radius, *r*_0_. The molecular mass (*M*_*m*_) and partial specific volume (*v*) of the monomer in solution were estimated using SEDNTERP (http://www.rasmb.org/sednterp/). The molecular mass for a monomer or trimer (*M*_*d/t*_) is a multiple of *M*_*m*_. *R* is the universal gas constant; *T* is the absolute temperature; *ρ* is the solvent density; ω is the angular velocity; and *ξ* is the baseline offset. With the known molecular weight of the single species, the oligomeric state can be determined. The program KaleidaGraph was used to fit these equations to the data. (C) Table includes the viral fusion protein, its family, and χ^2^ and *R* values for the curve fits shown in Fig. 2 at both pH 7 and 5. The single-species molecular weight (MW) is indicated for each SN-TM construct, and the best fit is indicated by an asterisk.

Previously, we demonstrated that paramyxovirus class I fusion protein TMDs, including those of PIV5 and HeV, self-associate in a monomer-trimer equilibrium in isolation, as determined via sedimentation equilibrium analytical ultracentrifugation (SE-AUC) (13). To determine whether TMD interactions are important beyond paramyxoviruses, fusion proteins from different viral families were analyzed. The viruses represent several major human pathogens, including Ebola virus, influenza virus, severe acute respiratory syndrome coronavirus (SARS CoV), and rabies virus. The Ebola virus fusion protein (GP), influenza virus HA, and SARS CoV spike protein (S) are class I viral fusion proteins. These proteins undergo a dramatic, irreversible structural rearrangement, so it is important to have mechanisms to maintain the prefusion conformation. Unlike class I fusion proteins, some class III fusion proteins, including rabies virus GP, can reverse the refolding process (14). Mutagenesis studies with SARS CoV S, Ebola virus GP, and influenza virus HA implicate the TMD in proper protein folding and function; however, none of the currently available crystal structures includes the TMD (5, 7, 15, 16). Utilizing the previously established SE-AUC system, chimeric proteins containing the TMD of interest fused with the protein staphylococcal nuclease (SN) were analyzed for oligomerization (Fig. 1A) (13, 17). The SN-TMD chimeric proteins were expressed in Escherichia coli, purified into detergent micelles, and density matched using deuterated water to negate any contribution to sedimentation by the micelle itself. It has been shown previously that the SN protein does not oligomerize (18). As a result of this preparation, any changes in sedimentation, as measured by absorbance, were the result of protein oligomerization (Fig. 1B).

**FIG 2:**
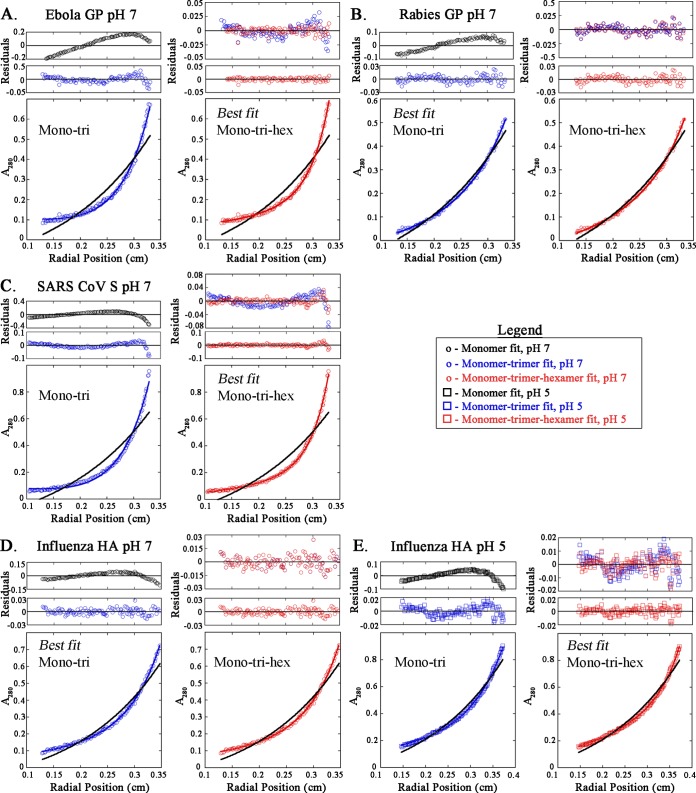
Trimeric association of TMDs of Ebola virus GP, SARS CoV S, rabies virus GP, and influenza virus HA, as determined by SE-AUC. Representative absorbance spectra for each SN-TMD are shown from analysis at 20,000 rpm. Absorbance data are plotted against normalized radial position and analyzed to determine best fit using χ^2^ and *R* values and residual plotting. Analysis is shown for the monomer-trimer (1:3) (blue) and monomer-trimer-hexamer (1:3:6) (red) curve fits. Best fit changed from pH 7 to 5 for influenza virus HA, so both sets of data are shown. Residuals are plotted above the absorbance spectra. The residuals for the monomeric fit are also shown in black. The monomeric curve fit is indicated by the black curve on each absorbance data plot. The residuals for the 1:3 (blue) and 1:3:6 (red) fits are overlaid to demonstrate the most appropriate fit. For example, the Ebola virus GP data points fit both 1:3 and 1:3:6 models according to the χ^2^ and *R* values; however, the residual plotting makes it apparent that the 1:3:6 fit is the most appropriate.

To determine the best model, the data points were fit to multiple models varying from monomer to multispecies fits, such as monomer-trimer-hexamer. Residual plotting and χ^2^ and *R* values were used to choose the best fit, and when more than one model was consistent with the data, the simplest model was chosen (Fig. 1C; see Fig. S1 in the supplemental material). Single-species analysis of each construct at pH 7 or 5 indicated a predicated molecular weight (N) that was greater than the molecular weight of the monomeric species (Fig. 1C). This suggests that the SN-TM proteins are of a higher oligomeric state or that multiple oligomeric species are present. The curve fit for each at 20,000 rpm is shown with the residuals plotted above (Fig. 2). Although influenza HA and rabies GP are fusion proteins of different classes (class I and class III, respectively), both best fit a monomer-trimer model, as the addition of a hexameric species did not improve residual distribution (Fig. 2B and D). The Ebola virus GP and SARS CoV S SN-TMDs were determined to best fit a monomer-trimer-hexamer model at pH 7. When the residuals for the monomer (Fig. 2, black circles) were plotted, the data points did not fit appropriately, unlike the 3-species model, which has evenly distributed residuals (Fig. 1C and 2). The presence of an additional oligomeric species not found for the other class I fusion proteins may be the result of association between SN-TMD trimers.

10.1128/mSphere.00047-18.1 FIG S1  (A) Construct design for SN-TM proteins with the C-terminal residues of the SN protein included. A short linker is found between the SN and TM sequences. The linker is from the original construct that utilized the glycophorin A TM domain, as well as some residues upstream from the TM. (B) TM sequence for each SN-TM construct is shown, with residue numbers indicated. χ^2^ and *R* values are shown for additional fits not shown in Fig. 2. Based on these values and residual plotting, these fits were determined to not be the most appropriate fits for the data obtained. Download FIG S1, TIF file, 0.2 MB. Copyright © 2018 Webb et al. 2018 Webb et al. This content is distributed under the terms of the Creative Commons Attribution 4.0 International license.

Low pH has been described as a trigger for many viral fusion proteins, including influenza virus HA and rabies virus GP. The rabies virus GP was found to require exposure to pH below 6.2 to drive membrane fusion, while influenza virus HA triggers in the acidic environment of an endosome (pH 6.0 to 6.5 in early endosomes and 4.5 to 5.5 in late endosomes) (19, 20). Though it has been shown that Ebola virus GP and SARS CoV S proteins require low pH for function, evidence suggests that this may be important for the cathepsin cleavage activity necessary to activate the fusion protein but not for their conformational changes (21–23). To determine whether association was affected by low pH, the SN-TMDs were prepared at pH 5 and analyzed via SE-AUC. The data points were fit to a monomer–*n*-mer curve (Fig. 1C) and then to multiple species curves. The SN-TMDs for the Ebola virus GP, influenza virus HA, and SARS CoV S at pH 5 best fit to a monomer-trimer-hexamer equilibrium, as determined by residual plotting, χ^2^, and *R* values (Fig. 2E; Fig. S2). Interestingly, the influenza virus HA SN-TMD protein data points exhibited a monomer-trimer equilibrium at pH 7, suggesting pH-induced changes in association. The rabies virus GP SN-TMD protein continued to exhibit a monomer-trimer equilibrium at both pHs. These results demonstrate that the fusion protein TMDs of various viral families self-associate, most typically as a trimer. The hexameric species detected are likely the result of two SN-TMD trimers interacting with one another. The Nipah virus F protein was recently found to oligomerize, forming higher-order species up to a hexamer of trimers (24). The Nipah virus F prefusion crystal structure revealed that six F trimers interacted in a ring structure that may contribute to prefusion protein stability. The close proximity of the fusion proteins in this tertiary structure could provide a platform for the interaction between trimeric TMDs. Additionally, an earlier study with influenza virus HA demonstrated that HA proteins located outside the site of contact were also important for membrane fusion. By interfering with the HA outsiders, membrane fusion was inhibited, suggesting a potential role for higher-order protein oligomerization in membrane fusion (21). Interestingly, the influenza HA SN-TMD fit a monomer-trimer equilibrium at pH 7 and then best fit a monomer-trimer-hexamer equilibrium at pH 5. The addition of a higher-order species at the acidic pH supports the idea that higher-order oligomerization could be important for membrane fusion and that the TMD interactions may contribute to the oligomerization. Direct analysis of the TMD has been limited, largely as a result of the inherent difficulty of working with such hydrophobic domains. Other systems have been used to study TMD dimerization, such as the TOXCAT system; however, these systems are unable to characterize higher-order oligomeric species (25). The data here demonstrate that trimeric TM-TM interactions occur for class I and III viral fusion proteins of different families. More importantly, these studies provide a tool to elucidate the residues that are critical for TM-TM association and, therefore, potentially critical for the proper folding and function of the full-length protein.

10.1128/mSphere.00047-18.2 FIG S2  Trimeric association of TMD of Ebola virus GP, SARS CoV S, and rabies virus GP at pH 5. Representative absorbance spectra for each SN-TMD from analysis at 20,000 rpm are shown. Curve fittings for the 1:3 (blue) and 1:3:6 (red) fits are shown, with the monomeric curve fits shown in black. Residuals are plotted above the absorbance spectra. The residuals for the 1:3 and 1:3:6 fits are overlaid to demonstrate the most appropriate fits. Download FIG S2, TIF file, 0.9 MB. Copyright © 2018 Webb et al. 2018 Webb et al. This content is distributed under the terms of the Creative Commons Attribution 4.0 International license.
