# Supplementary figures and images for: Transmembrane Domains of Highly Pathogenic Viral Fusion Proteins Exhibit Trimeric Association In Vitro
Source: mSphere. 2018 Apr 18;3(2):e00047-18. doi: 10.1128/mSphere.00047-18 (PMC5907656; doi:10.1128/mSphere.00047-18)

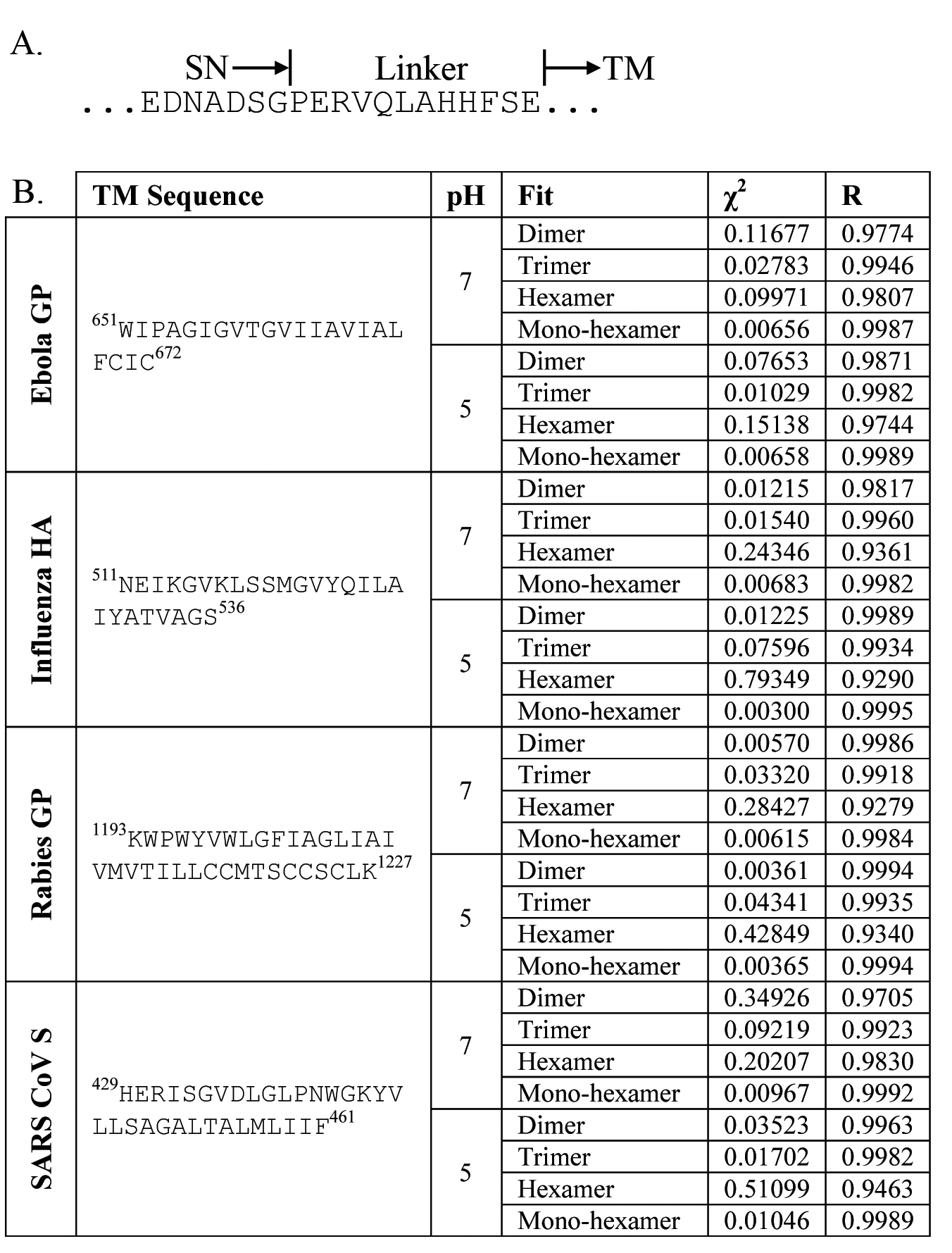

Supplement: FIG S1 [file sph002182516sf1.tif]

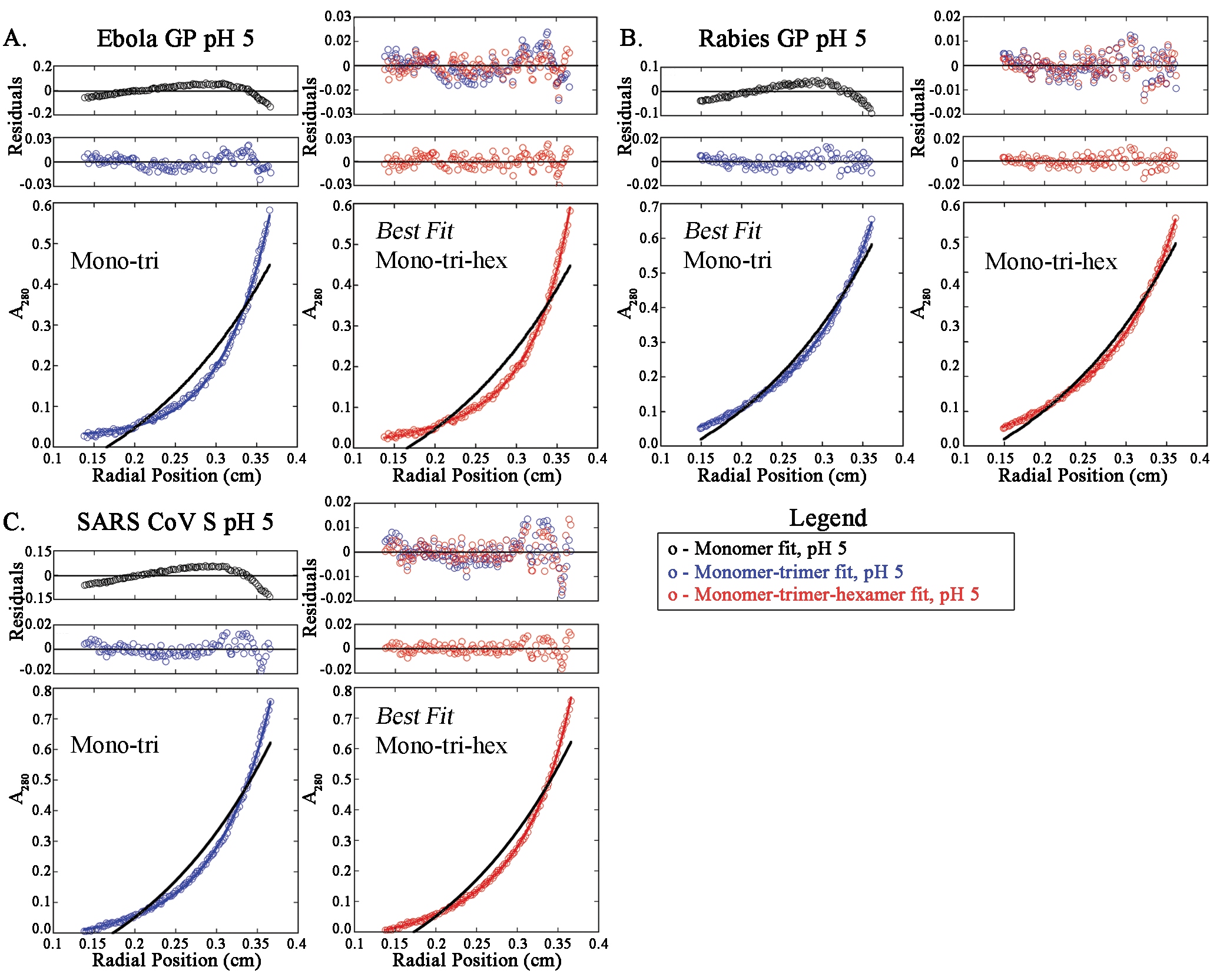

Supplement: FIG S2 [file sph002182516sf2.tif]
